# Photosynthetic Responses to Rising CO_2_ and Temperature: Mechanisms, Models, and Field Evidence

**DOI:** 10.1111/gcb.71076

**Published:** 2026-08-28

**Authors:** Carl J. Bernacchi, Amanda P. Cavanagh

**Affiliations:** ^1^ Department of Crop Sciences University of Illinois Urbana–Champaign Urbana Illinois USA; ^2^ School of Life Sciences University of Essex Colchester UK

## Abstract

Combined increases in atmospheric CO_2_ and warming temperatures impact photosynthesis in complex yet mechanistically predictable ways. Seminal work in this area showed that temperatures above a thermal optimum reduce photosynthesis primarily by increasing photorespiration, but that elevated CO_2_ and higher temperature can act synergistically to raise the thermal optimum of photosynthesis and increase absolute photosynthetic rates. The modeling work that led to this prediction outlined both the synergistic effects of CO_2_ and temperature and hypothesized scenarios that could deviate from theory. Here, the assumptions underlying models of CO_2_ × temperature interactions in photosynthesis are reviewed, with emphasis on advances in in vivo enzyme kinetics, diffusive limitations, photosynthetic acclimation, and atmospheric feedbacks. Advances in Rubisco biochemistry, mesophyll conductance, field experiments, and canopy‐scale modeling show why photosynthetic responses under future climates often deviate from the theoretical CO_2_ × temperature response. Scaling from leaf‐level physiology to canopy carbon uptake is addressed to identify priorities for next‐generation experiments and models. Together, this review highlights the work of the late Prof. Stephen P. Long, beginning with his pioneering manuscript that first addressed the synergistic effects of CO_2_ and temperature on photosynthesis.

## Graphical abstract

This review revisits the mechanistic basis of CO_2_ × temperature interactions in C_3_ photosynthesis and evaluates how subsequent research has refined this framework. We synthesize evidence showing how acclimation, diffusive limitations, and canopy processes modify photosynthetic responses to rising CO_2_ and warming across scales.

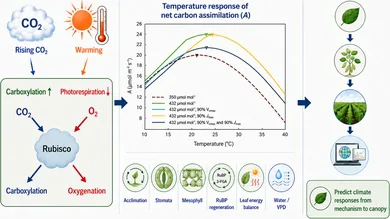

## 1 Introduction

The rise in global temperature driven primarily by increasing atmospheric carbon dioxide (CO_2_) concentrations from anthropogenic activities has been recognized for decades and shows no evidence of slowing (Calvin et al. 2023; Hansen et al. 1988; Houghton et al. 1998). Vegetation responds directly to temperature, which regulates metabolic processes, and CO_2_, which governs photosynthetic carbon assimilation. Because photosynthesis responds interactively to temperature and CO_2_, predicting vegetation responses to concurrent changes in both factors is critical for evaluating impacts on crop production, understanding the dynamics of natural ecosystems, and assessing feedbacks between terrestrial carbon cycling and atmospheric CO_2_ concentrations (Long 1991).

Early experimental and modeling studies often represented CO_2_ enrichment and temperature independently. The temperature response of photosynthesis was described as increasing to a thermal optimum (T_opt_) followed by a decline at supra‐optimal temperatures (Berry and Bjorkman 1980) and rising CO_2_ was expected to stimulate photosynthesis without changing the shape of the temperature response curve. Empirical evidence for interactions between CO_2_ and temperature was emerging (Badger et al. 1982), yet many experimental frameworks still represented these effects as additive rather than mechanistically coupled (Badger et al. 1982).

A key conceptual advance in understanding the interaction between rising CO_2_ and temperature was provided by Long (1991). Drawing on the Farquhar–von Caemmerer–Berry (FvCB) model of C_3_ photosynthesis (Farquhar et al. 1980; Farquhar and von Caemmerer 1982), Long showed that rising CO_2_ modifies the temperature response of assimilation by altering the balance between carboxylation and oxygenation of the primary carbon‐assimilating enzyme ribulose‐1,5‐bisphosphate (RuBP) carboxylase/oxygenase (Rubisco). Carboxylation leads to net carbon gain via the photosynthetic carbon reduction cycle, whereas oxygenation initiates photorespiration resulting in carbon loss. The relative rates of these competing processes are strongly temperature dependent and, while empirical evidence had established their importance, the mechanisms were not yet fully integrated into models used to predict plant responses to climate change (Long 1991).

Since the publication of Long (1991), a substantial body of work has both tested and extended the interaction between CO_2_ and temperature. Studies across a wide range of species and environments confirmed that the stimulation of photosynthesis by elevated CO_2_ increases with temperature and can shift the thermal optimum of carbon assimilation. This work cemented the interaction between CO_2_ and temperature as a critical component of plant physiological responses to climate change. Subsequent work contributed to this understanding, including biochemical acclimation of photosynthetic capacity through adjustments in Rubisco content and electron transport, the influence of resource availability, and coupled environmental stresses that further influence CO_2_ and temperature responses (Ainsworth and Long 2005; Ainsworth and Rogers 2007; Bernacchi et al. 2001, 2003; Dusenge et al. 2019; Leakey et al. 2009; Sage 1994; Yamori et al. 2014). Long (1991) also stimulated efforts to scale these interactions from the leaf to canopy and ecosystem levels, leading to their incorporation into models of plant and ecosystem function (McMurtrie and Wang 1993; Amthor 1994; Bernacchi et al. 2013; de Pury and Farquhar 1997; Drewry et al. 2010a, 2010b; Rogers et al. 2017). Together, this body of work reflects the enduring influence of Long (1991) in shaping how vegetation responses to climate change are conceptualized, measured, and modeled.

Here, we revisit progress on five key research areas that build upon the work presented in Long (1991): the biochemical basis of the CO_2_ × temperature interaction; improved parameterization of Rubisco‐limited and RuBP‐regeneration‐limited photosynthesis; recognition of acclimation, stomatal conductance, and mesophyll conductance as dynamic modifiers of the realized response; scaling of leaf‐level processes to canopy carbon gain; and evidence‐based opportunities to improve photosynthetic performance under warming through enhanced RuBP regeneration and reduced photorespiratory losses.

## 2 Foundation of the CO_2_ × Temperature Interaction

The interaction between CO_2_ and temperature described by Long (1991) was grounded in the biochemical properties of Rubisco and their representation in the FvCB model of C_3_ photosynthesis (Farquhar et al. 1980). The basis of the model is that net carbon assimilation is represented as:

(1) $$A=V_{c}-0.5V_{o}-R_{d}$$

where *V*_*c*_ and *V*_*o*_ are the rates of carboxylation and oxygenation of Rubisco, respectively; *R*_*d*_ is the rate of mitochondrial respiration in the light; and the constant 0.5 represents the amount of CO_2_ released for each oxygenation event (Farquhar et al. 1980). The ratio of *V*_*c*_ to *V*_*o*_ varies predictably based on environmental conditions and is represented as the photosynthetic CO_2_ compensation point, Γ*, (e.g., Walker and Ort 2015). Equation (1) can therefore be rewritten as:

(2) $$A=V_{c}\left(1-\Gamma^{*}/C\right)-R_{d}$$

where *C* is the CO_2_ concentration at the site of carboxylation. Net assimilation is determined by the minimum of the Rubisco‐limited (*v*_*c*_) and RuBP‐regeneration‐limited (*v*_*j*_) rates:

(3) $$A=\min\left\{v_{c},v_{j}\right\}\left(1-\Gamma^{*}/C\right)-R_{d}$$

The Rubisco‐ and RuBP‐regeneration‐limited rates are represented as follows:

(4) vc=Vc,max·CC+Kc1+OKo

(5) $$v_{j}=\frac{J\cdot C}{4.6C+10.8\Gamma^{*}}$$

where *V*_*c,max*_ is the maximum rate of carboxylation, *K*_*c*_ and *K*_*o*_ are the Michaelis constants for carboxylation and oxygenation, respectively, *O* is the oxygen concentration, and *J* is the rate of electron transport through photosystem II. A third limitation, triose phosphate utilization‐limited photosynthesis ($v_{p}$), was not included in the original formulation of the model. Although this limitation can be significant under some conditions (e.g., McClain and Sharkey 2019), predicting when it becomes relevant remains difficult, making its inclusion in general model applications challenging.

At the enzyme scale, temperature and CO_2_ alter the balance between Rubisco carboxylation and oxygenation through effects on catalytic kinetics, substrate availability, and photorespiratory carbon loss. At the leaf scale, these biochemical processes are expressed through the competing limitations imposed by Rubisco carboxylation capacity (i.e., *V*_*c,max*_) and RuBP regeneration capacity (i.e., *J*_*max*_). Therefore, establishing this biochemical foundation is necessary for understanding why elevated CO_2_ changes not only the magnitude of photosynthesis but also the shape and thermal optimum of the photosynthetic temperature response.

### 2.1 Basics of Rubisco Responses to Temperature and CO_2_

**FIGURE 1:**
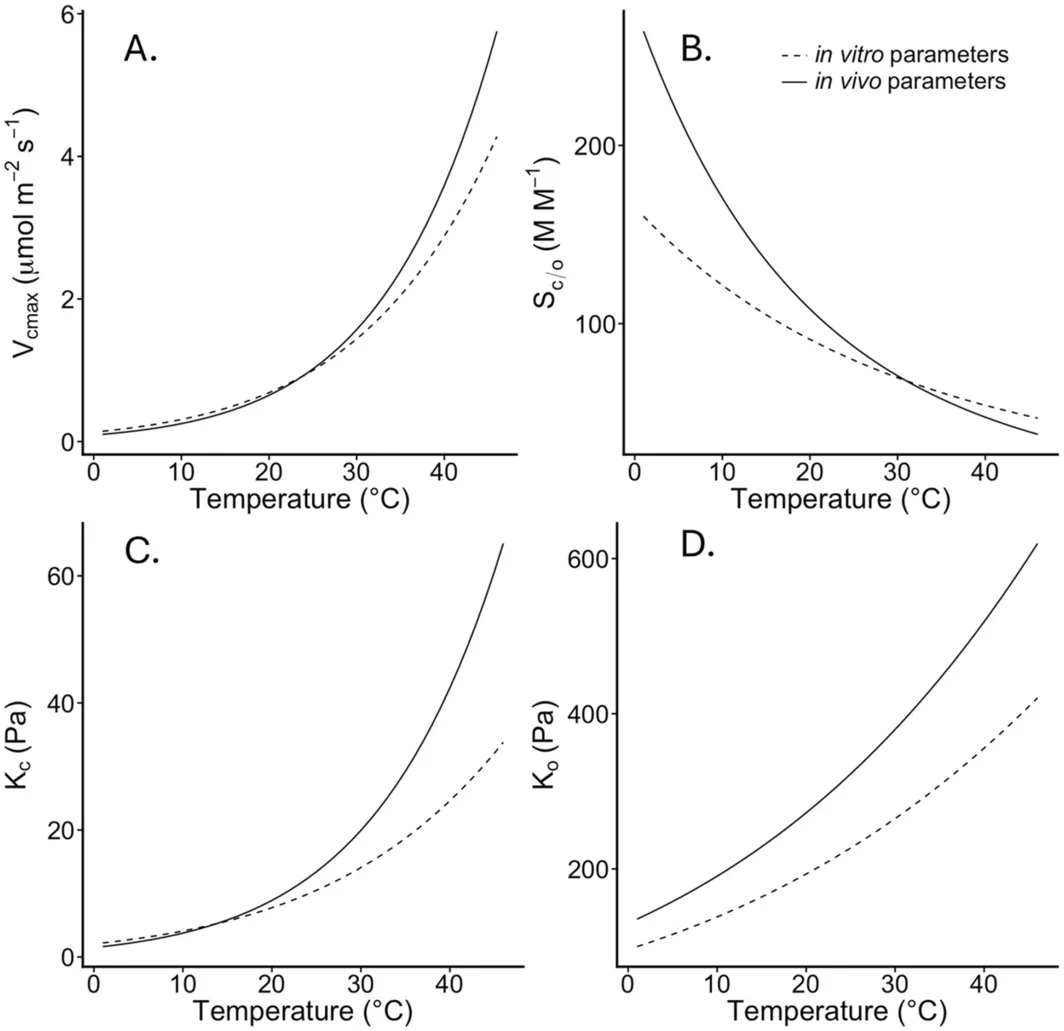
Temperature responses of tobacco Rubisco kinetic parameters derived in vitro from leaf extracts (dashed line, Cavanagh 2016), or in vivo from Rubisco antisense using leaf gas exchange (solid line, Bernacchi et al. 2001). Shown are the maximum Rubisco carboxylation rate (*V*_*c,max*_), normalized to unity at 25°C (A); Rubisco specificity for CO_2_ relative to O_2_ (S_c/o_; B); and the Michaelis constants for carboxylation (*K*_*c*_; C) and oxygenation (*K*_*o*_; D).

The interaction between temperature and CO_2_ in C_3_ photosynthesis arises from the dual function of Rubisco, which catalyzes both the carboxylation and oxygenation of RuBP. The carboxylation reaction produces two molecules of 3‐phosphoglycerate (3‐PGA) that enter the Calvin‐Benson cycle, whereas oxygenation produces one molecule of 3‐PGA and one molecule of 2‐phosphoglycolate (2‐PG), which cannot be metabolized via the Calvin‐Benson cycle. Instead, photorespiration salvages 75% of the carbon from 2‐PG into 3‐PGA via a series of enzymatic conversions and transport steps spanning the chloroplast, peroxisome, and mitochondria. Because the remaining 25% of carbon is released as CO_2_ and photorespiration requires ATP and NADPH from the light‐dependent reactions, it significantly reduces C_3_ photosynthetic efficiency (Peterhansel et al. 2010; Walker et al. 2016). As temperatures increase, Rubisco catalytic turnover rates for both reactions increase through the expected Arrhenius response of enzyme‐catalyzed reactions (Badger 1977; Moore et al. 2021). However, the relative rates of oxygenation increase more rapidly with temperature because of declines in both Rubisco's substrate specificity for CO_2_ versus O_2_ (*S*_*C/O*_) and the solubility of CO_2_ relative to O_2_ (Jordan and Ogren 1984). Rubisco's substrate specificity reflects the ratio of catalytic efficiencies for carboxylation and oxygenation accounting for both substrate binding (i.e., effective Michaelis–Menten constants *K*_*C*_ and *K*_*O*_) and turnover rates (${k\mathrm{cat}}_{{\mathrm{CO}}_{2}}$ and ${k\mathrm{cat}}_{O_{2}}$) (Laing et al. 1974). The effective Michaelis constant for CO_2_ increases more strongly with temperature than that for O_2_, reducing the relative catalytic efficiency of carboxylation (Figure 1; Boyd et al. 2019). The resulting decline in *S*_*C/O*_ accounts for ~68% of the temperature dependence of the decline in carboxylation relative to oxygenation, while reduced CO_2_ solubility accounts for the remaining ~32% (Jordan and Ogren 1984; Long 1991). Rising temperature and atmospheric CO_2_ therefore impose opposing influences on Rubisco oxygenation and subsequent photorespiratory losses. Higher temperature lowers Rubisco specificity and favors oxygenation, whereas higher CO_2_ suppresses oxygenation favoring carboxylation. Because atmospheric CO_2_ enrichment is coupled to warming, projected increases in growing‐season temperature are expected to sustain or increase photorespiratory flux even under end‐of‐century CO_2_ concentrations (Cavanagh and Ort 2023; Walker et al. 2016). Rubisco specificity therefore remains central to the temperature response of photosynthesis under moderate sustained warming, while Rubisco activation state, maintained by Rubisco activase, becomes increasingly important as temperatures approach and exceed the thermal optimum (Scafaro et al. 2023).

Above the thermal optimum, declines in photosynthesis increasingly reflect co‐limitation by Rubisco activation state, chloroplast electron transport, and the capacity of the Calvin‐Benson cycle to regenerate RuBP (Bernacchi et al. 2025; Moore et al. 2021; Scafaro et al. 2023). At the leaf scale, these biochemical processes are represented by two limiting rates of photosynthesis: (1) the capacity for Rubisco carboxylation is described by the maximum carboxylation rate, *V*_*cmax*_, which integrates Rubisco catalytic properties with enzyme concentration and activation state, and (2) The capacity for RuBP regeneration is described by the maximum rate of electron transport, *J*_max_, which reflects both Calvin–Benson cycle capacity and the supply of ATP and NADPH from the light‐dependent reactions. Declining electron transport rates reflect heat damage to PSII and thylakoid membranes, together with reduced chlorophyll content and impaired ATP production (Allakhverdiev et al. 2008; Prasad et al. 2017; Wise et al. 2004). Electron transport and RuBP regeneration are linked because the ATP and NADPH generated by the photosynthetic electron transport chain are required to drive the regenerative reactions of the Calvin–Benson cycle. However, RuBP regeneration also depends on the catalytic capacity of the Calvin–Benson cycle enzymes themselves, and the thermotolerance of these remains relatively poorly explored (Moore et al. 2021; Raines 2022). Increased photorespiratory flux consumes a larger proportion of ATP and NADPH as temperatures increase, shifting photosynthetic control from a primary Rubisco limitation under moderate warming toward increasing RuBP‐regeneration limitation at higher temperatures.

### 2.2 The Long (1991) Model Predictions for CO_2_ × Temperature Interactions

The FvCB model establishes Rubisco carboxylation, oxygenation, and RuBP regeneration in a physiological context, where net leaf assimilation can be modeled as the minimum of the Rubisco‐ and RuBP‐regeneration‐limited photosynthetic rates (Farquhar et al. 1980). Applying this model, Long (1991) showed that elevated CO_2_ changes the temperature response of C_3_ photosynthesis such that the greatest photosynthetic stimulation occurs at temperatures above T_opt_, where photorespiratory losses are greatest. By contrast, most models up to this point had treated CO_2_ as a factor that increased leaf‐level photosynthesis by a fixed amount. Three predictions from this analysis provided the foundation for subsequent work. First, the stimulation of photosynthesis by elevated CO_2_ should increase with temperature because photorespiration increases as temperature rises and higher CO_2_ suppresses the oxygenation reaction that drives that loss. Second, elevated CO_2_ should raise the thermal optimum of C_3_ photosynthesis because suppression of photorespiration allows assimilation to remain higher at warmer temperatures before limitations imposed by RuBP regeneration, electron transport, respiration, or enzyme deactivation dominate. Third, the interaction between CO_2_ and temperature should influence canopy carbon gain because elevated CO_2_ changes the quantum yield and light compensation point of individual leaves. Because respiration in the dark increases with temperature, the light compensation point also rises with temperature at ambient CO_2_, such that shaded leaves are likely to respire rather than assimilate carbon. At elevated CO_2_, these changes alter the proportion of the canopy operating above the light compensation point, particularly among shaded leaves, and therefore modify the integrated canopy response to temperature.

An assumption of many modeling applications at the time was that Rubisco kinetic parameters measured under controlled in vitro conditions could be used to represent Rubisco function in intact leaves. Purified enzyme assays provided the clearest estimates of *K*_C_, *K*_O_, S_C/O_, and *K*_cat_, but these assays did not reproduce the chloroplast environment in which Rubisco operates (Figure 1). In vivo, Rubisco activity is influenced by substrate gradients, stromal pH and ionic composition, metabolite pools, macromolecular crowding, and regulation of activation state (Amaral et al. 2024; Bernacchi et al. 2002; Carmo‐Silva et al. 2015; Hayer‐Hartl and Hartl 2020; Kirchhoff 2019; Lobo et al. 2024; Mott and Berry 1986). Long (1991) also treated Rubisco specificity as a stable catalytic property, acknowledging that acclimation to elevated CO_2_ altered photosynthetic capacity through reductions in Rubisco amount or activation state, or changes in allocation between *V*_*c,max*_ and *J*_*max*_. Recent work indicates that growth temperature may also alter Rubisco biochemistry through changes in holoenzyme composition, including differential expression of small‐subunit isoforms, with associated effects on catalytic behavior (Askey et al. 2026; Cavanagh et al. 2023; Martin‐Avila et al. 2020). Thus, Long's assumption that CO_2_ acclimation would not necessarily alter Rubisco specificity remains broadly useful, while newer evidence suggests that thermal acclimation can modify enzyme properties that were treated as fixed in early model applications.

## 3 Testing and Refining the Assumptions of Long (1991)

The framework proposed by Long (1991) captured the fundamental interaction between CO_2_ and temperature but relied on key assumptions about parameterization of the FvCB model and plant physiology. These assumptions have been tested and refined in subsequent work. Specifically, advances in in vivo parameterization and the integration of models with experimental observations have revealed how physiological acclimation alters key parameters governing carbon assimilation under elevated CO_2_ and warming.

### 3.1 Improving Model Parameters

As originally parameterized, the leaf‐level model of photosynthesis relied on kinetic parameters of Rubisco derived from in vitro enzyme assays. These estimates varied widely and were obtained under conditions that did not reflect the biochemical environment of the chloroplast (Figure 1; von Caemmerer et al. 1994). Nevertheless, these parameters were widely used in modeling, including in the analysis applied by Long (1991), under the assumption that in vitro Rubisco kinetics adequately represent in vivo behavior (Diaz‐Espejo 2013).

This assumption was tested using two key advances: antisense suppression of the Rubisco small subunit (anti‐rbcS) and improvements in gas exchange methodology (von Caemmerer et al. 1994). In anti‐rbcS tobacco, Rubisco content was reduced to approximately 34% of wild‐type levels, resulting in Rubisco‐limited photosynthesis over a much broader range of CO_2_ concentrations (von Caemmerer et al. 1994). In contrast, wild‐type plants typically transition to RuBP‐regeneration limitation at relatively low CO_2_, restricting the range over which Rubisco kinetics can be inferred. The expanded limitation range in anti‐rbcS plants enabled more robust estimation of kinetic parameters, particularly when combined with precise control of environmental conditions in modern gas exchange systems. These approaches demonstrated that in vivo Rubisco kinetic parameters are often at the lower end of values derived from in vitro measurements, with important implications for model parameterization (Figure 1; von Caemmerer et al. 1994).

Advances in quantifying the temperature dependence of Rubisco kinetics have further refined model predictions. Using similar anti‐rbcS approaches, gas exchange measurements conducted across a wide range of temperatures improved both the characterization of the temperature responses of key kinetic parameters and the ability to model photosynthesis under variable environmental conditions (Bernacchi et al. 2001; Walker et al. 2013). However, despite these advances, in vivo kinetic data remain limited to relatively few species, constraining broader generalization across plant functional types (Galmés et al. 2016; Walker et al. 2013).

As with Rubisco‐limited photosynthesis, the parameterization of the RuBP‐regeneration‐limited portion of the model has also advanced since the original analysis of Long (1991). Whereas the Rubisco‐limited component is based on enzyme kinetics, the RuBP‐regeneration‐limited component reflects the balance between the biochemical demand for ATP and NADPH and the supply of electrons through photosynthetic electron transport, typically represented as a nonlinear function of irradiance (Farquhar et al. 1980; Long and Bernacchi 2003). The integration of gas exchange with chlorophyll fluorescence enabled direct estimation of the temperature responses of parameters related to RuBP regeneration, providing improved constraints on electron transport processes under varying environmental conditions (Bernacchi et al. 2003). Advances in mechanistic understanding of electron transport have improved model performance (June et al. 2004; Johnson and Berry 2021) and have led to further improvements related to the temperature response of photosynthesis. These developments demonstrate, as originally outlined in Long (1991), that accurate parameterization is essential for predicting both the magnitude and temperature sensitivity of photosynthesis under future conditions, and that uncertainty in these parameters remains a primary source of divergence among model projections (Johnson and Berry 2021).

### 3.2 Bridging Models and Measurements

**FIGURE 2:**
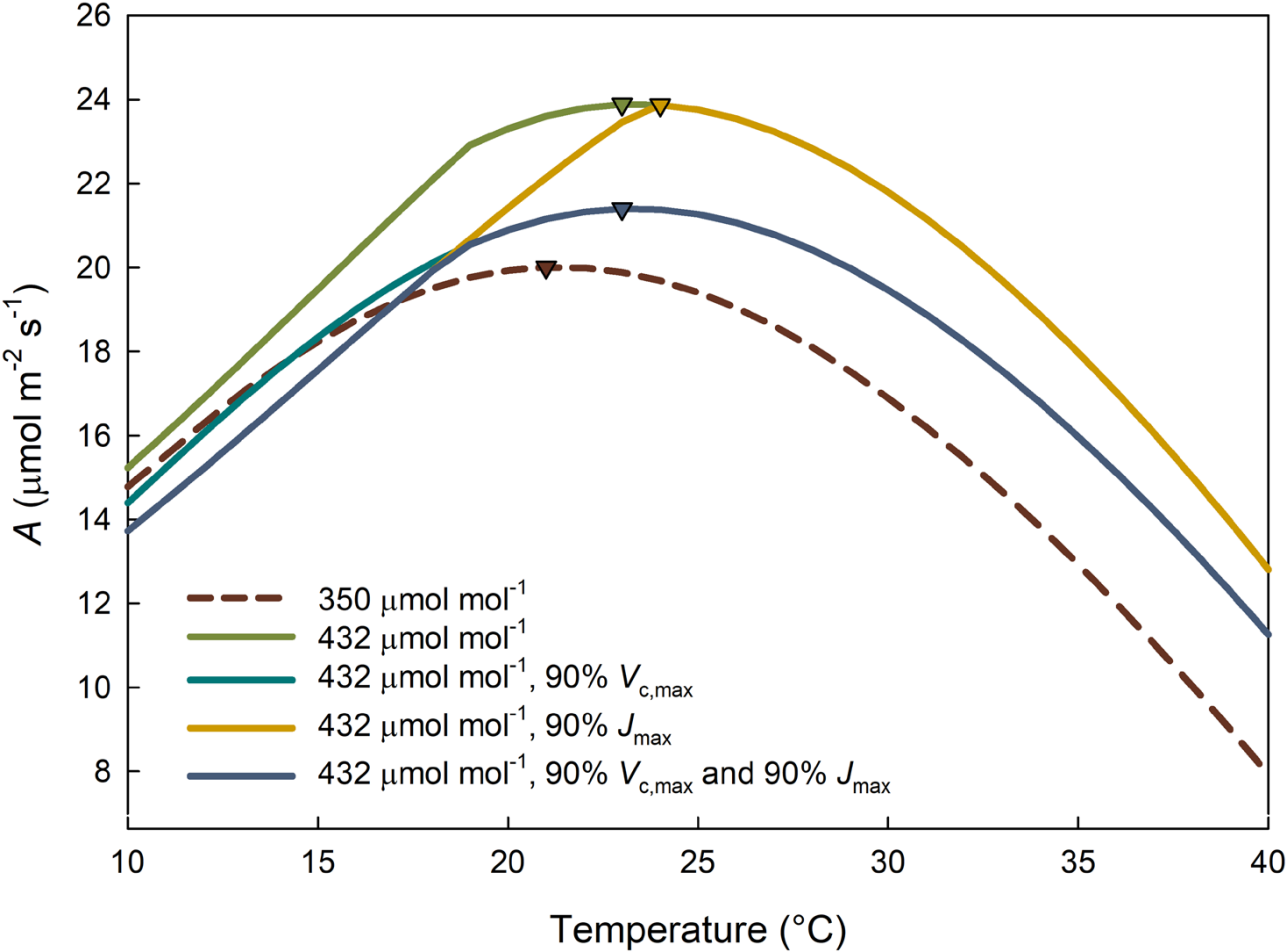
Temperature response of net light‐saturated carbon assimilation (*A*) modeled at ambient atmospheric CO_2_ concentrations in 1991 and 2026. Included are modeled scenarios in which *V*_*c,max*_ and/or *J*_*max*_ are reduced by 10% to represent photosynthetic acclimation. Outputs were modeled using the equations presented in Long (1991) with the temperature‐response functions required for modeling based on Bernacchi et al. (2001) for Rubisco‐limited photosynthesis (e.g., Equations (2) and (4)) and Bernacchi et al. (2003) for RuBP‐regeneration‐limited photosynthesis (e.g., Equations (2) and (5)).

In the 35 years since Long (1991), atmospheric CO_2_ concentrations have risen from approximately 350 μmol mol^−1^ to 432 μmol mol^−1^. Following this increase, photosynthetic rates at a modeled thermal optimum of 21°C are estimated to have increased by approximately 18%, from approximately 20 to 23.6 μmol m^−2^ s^−1^, assuming that the *V*_*c,max*_ and *J*_*max*_ values used in Long (1991) have remained fixed (Figure 2). As temperatures rise above the thermal optimum, the higher thermal optimum of plants grown under elevated CO_2_ increases the percentage stimulation of photosynthesis (Figure 2).

Long (1991) framed acclimation not simply as a reduction in photosynthetic capacity, but as a process that could reshape the temperature dependence of CO_2_ responses. His work demonstrated that acclimation could alter the theoretical CO_2_ × temperature response, while subsequent work has clarified how variable acclimation responses are among species and growth environments. Prior to Long (1991), studies had shown that growth under elevated CO_2_ could reduce Rubisco activation state and/or Rubisco content by 0%–60% across species (Campbell et al. 1988; Rowland‐Bamford et al. 1991; Sage et al. 1989). Long incorporated this acclimation response into model simulations by reducing *V*_*c,max*_ by 20% and 40%. Those simulations showed that photosynthesis at elevated CO_2_ of 650 μmol mol^−1^ generally remained higher than at 350 μmol mol^−1^ across much of the temperature range, even when *V*_*c,max*_ was reduced by 40%. Furthermore, even with simulated acclimation responses, elevated CO_2_ shifted the thermal optimum upward. However, the simulations also revealed that at lower temperatures acclimation could diminish, or under some conditions reverse, the photosynthetic benefit of elevated CO_2_.

The increase in atmospheric CO_2_ since 1991 provides a useful context for evaluating how acclimation might modify the modeled photosynthetic gains. Compared with a no‐acclimation scenario, a 10% reduction in *V*_*c,max*_ and/or *J*_*max*_ leads to either no change in or a reduction in photosynthesis (Figure 2). Compared with modeled photosynthetic–temperature responses under atmospheric CO_2_ concentrations in 1991, photosynthetic rates under current CO_2_ are higher across most temperatures, except below the thermal optimum. These simulations emphasize that acclimation can alter both the magnitude of the modeled CO_2_ response and the temperature range over which elevated CO_2_ provides a photosynthetic advantage.

**FIGURE 3:**
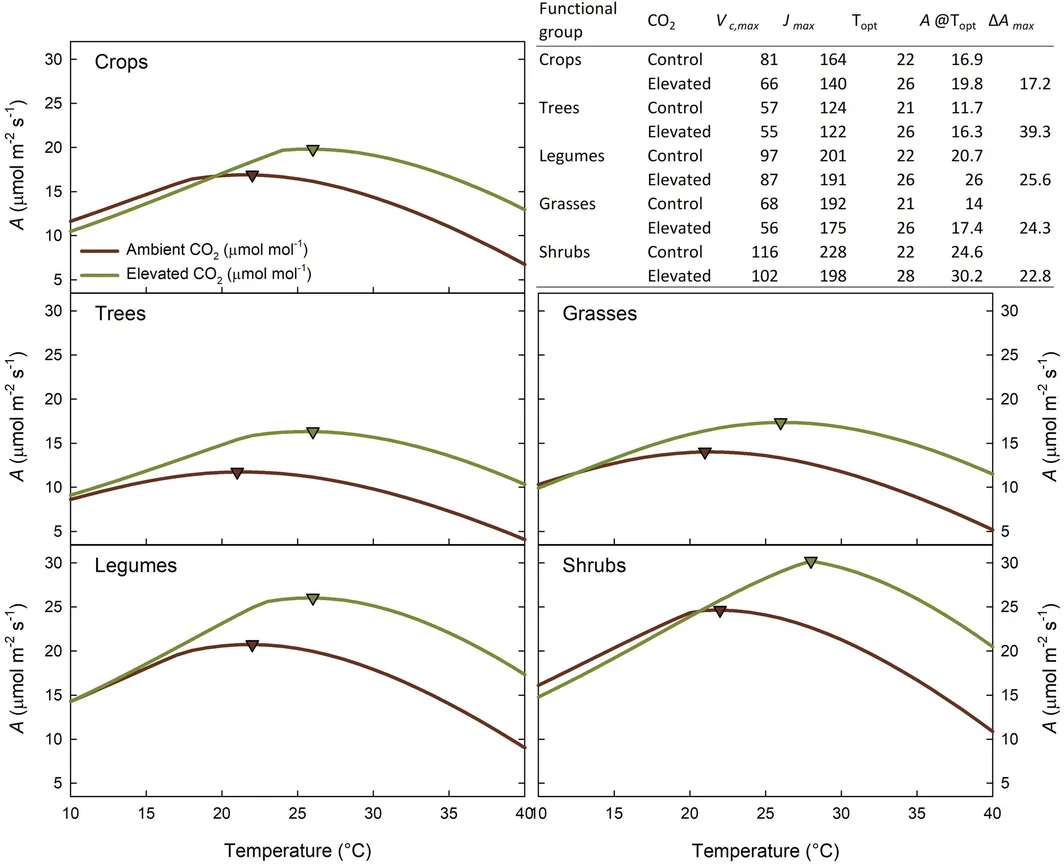
Temperature response curves of light‐saturated net carbon assimilation (*A*) modeled using the FvCB model for different plant functional types at current ambient and elevated CO_2_ concentrations. Curves were modeled based on the values of *V*_*c,max*_ and *J*_*max*_ from Ainsworth and Rogers (2007) shown in the inset table. Down‐facing triangles in each panel represent the thermal optimum of photosynthesis (T_opt_). Included in the inset table are the values of T_opt_, the photosynthetic rate at T_opt_ (A@T_opt_), and the percentage increase in maximum photosynthesis (Δ*A*_*max*_). Note that the values of *V*_*c,max*_ and *J*_*max*_ include photosynthetic acclimation to CO_2_ but not to temperature.

Experimental observations demonstrate that photosynthetic responses to elevated CO_2_ and temperature are highly variable among species and are modified by physiological acclimation to the growth environment (Ainsworth and Long 2005; Ainsworth and Rogers 2007; Kumarathunge et al. 2019; Medlyn et al. 1999; Sage et al. 1989; Way and Yamori 2014; Bernacchi et al. 2013). This is evident when modeling photosynthetic temperature response curves (Figure 3) using *V*_*c,max*_ and *J*_*max*_ values under ambient and elevated CO_2_ for various plant functional types provided in a meta‐analysis (Ainsworth and Rogers 2007). Depending on plant functional type, elevated CO_2_ can decrease *V*_*c,max*_, *J*_*max*_, or both (Ainsworth and Rogers 2007). This variation may reflect coordinated differences in several traits, including Rubisco catalytic properties, Rubisco activase thermotolerance, the allocation of leaf nitrogen among photosynthetic processes, leaf anatomy, stomatal and hydraulic strategies, and source–sink balance. Broad plant functional‐type categories summarize observed variation but do not necessarily provide a mechanistic explanation. Resolving these mechanisms will require coordinated experiments across species to quantify these traits using standard or high‐throughput methods (e.g., Fu et al. 2022). The modeled curves demonstrate the diversity of temperature response curve shapes and the extent to which altered growth environments can modify those curves (Figure 3). Growth at elevated temperature can similarly induce acclimation responses that can alter photosynthetic temperature responses (Bagley et al. 2015; Hikosaka et al. 2006; Moore et al. 2021; Rosenthal et al. 2014; Way and Yamori 2014). These findings underscore that model parameters are dynamic properties shaped by species, plant functional types, and environmental growth conditions.

Field‐based CO_2_ enrichment and warming experiments have provided critical tests of whether the modeled CO_2_ × temperature responses are realized under environmental conditions. Free‐Air CO_2_ Enrichment (FACE) experiments generally support the expectation that elevated CO_2_ stimulates photosynthesis and carbon gain (Ainsworth et al. 2003; Ainsworth and Long 2021; Long et al. 2006; Norby 2025; Norby and Zak 2011), but they also show that the magnitude and persistence of this stimulation depend on factors such as nitrogen availability, water status, sink strength, canopy structure, and seasonal dynamics. Temperature manipulation experiments similarly demonstrate that warming can alter photosynthetic capacity and thermal optima (Bernacchi et al. 2023, 2025; Cui et al. 2026; Kumagai et al. 2022; Wu et al. 2025), but responses often depend on whether warming is accompanied by changes in vapor pressure deficit, soil moisture, and resource availability. At the whole‐plant scale, however, evidence that the CO_2_ × temperature interaction increases growth remains less conclusive. A model‐oriented meta‐analysis of woody plants found insufficient evidence for a consistent increase in the growth response to elevated CO_2_ with temperature, potentially because photosynthetic acclimation, resource limitation, sink strength, and/or treatment duration uncoupled leaf‐level responses from biomass accumulation (Baig et al. 2015). Revisiting these experiments to determine whether the magnitude of the growth interaction covaried with photosynthetic acclimation would be a valuable future research direction. Increasingly, field observations have become essential for constraining *V*_*c,max*_, *J*_*max*_, and acclimation functions in models, while also revealing model‐data discrepancies that cannot be resolved by leaf biochemistry alone.

These findings reinforce the framework established by Long (1991) while emphasizing that accurate prediction of future photosynthetic responses requires representation of acclimation processes, plant functional diversity, and environmental context. However, improved representation of biochemical parameterization and acclimation is insufficient if models do not also capture the diffusive constraints that determine CO_2_ supply to the chloroplast.

## 4 Diffusive Limitations on Photosynthesis and Influence on Model Performance

The interaction between CO_2_ and temperature depends not only on Rubisco kinetics and RuBP regeneration, but also on diffusion of CO_2_ from the atmosphere to the site of carboxylation. The role of stomata in regulating CO_2_ supply to the leaf was already established (Jones 1985), as were general stomatal responses to environmental conditions (Ball et al. 1987). However, many model applications at the time effectively assumed that mesophyll conductance was infinite so that chloroplastic CO_2_ concentration (*C*_c_) was similar to intercellular CO_2_ concentration (*C*_i_; von Caemmerer et al. 2025). Because Rubisco carboxylation and oxygenation are governed by *C*_c_ rather than atmospheric or intercellular CO_2_, the CO_2_ × temperature response depends on the combined effects of stomatal conductance, which regulates CO_2_ entry into the leaf, and mesophyll conductance, which involves transfer from the intercellular airspaces to the chloroplast. Because both conductances respond to environmental conditions (e.g., Bernacchi et al. 2002), they can modify the apparent biochemical response of photosynthesis and influence model performance under future climate conditions. Resolving how stomatal and mesophyll conductance regulate *C*_c_ is essential to accurately predict photosynthetic responses to elevated CO_2_ and warming.

### 4.1 Stomatal Conductance and Temperature

Stomatal conductance represents the first major diffusional control on photosynthesis by regulating the exchange of CO_2_ and water vapor between the atmosphere and the leaf (Lawson and Matthews 2020). Instantaneous stomatal responses to environmental conditions have provided the basis for empirical and semi‐mechanistic models of stomatal conductance (Ball et al. 1987; Leuning 1995). Incorporating stomatal conductance into biochemical photosynthesis models is challenging because stomatal behavior and photosynthetic carbon assimilation are interdependent: stomata influence intercellular CO_2_ concentration, while photosynthetic demand influences the CO_2_ gradient that helps regulate stomatal behavior. Long (1991) avoided this computational challenge by prescribing *C*_*i*_/*C*_*a*_ as an empirical function of temperature rather than explicitly coupling a stomatal‐conductance model to photosynthesis. This approach differs from most contemporary stomatal models, in which *g*_*s*_ is coupled to assimilation and responds to vapor pressure deficit or relative humidity rather than directly to temperature. The formulation represented the effect of temperature on CO_2_ supply without resolving the effects of atmospheric demand or leaf energy balance on stomatal behavior.

Elevated CO_2_ commonly reduces stomatal conductance as it allows leaves to maintain higher intercellular CO_2_ concentrations while reducing transpiration (Lawson and Leakey 2024). Manipulative experiments have demonstrated that growth under elevated CO_2_ often reduces stomatal conductance (Bernacchi et al. 2006; Tricker et al. 2005; Wall et al. 2000), whereas evidence suggests limited or no acclimation of stomatal sensitivity to elevated CO_2_ (Leakey et al. 2006; Xu et al. 2016). However, a meta‐analysis of stomatal responses to CO_2_ shows that *C*_*i*_/*C*_*a*_ remains approximately conserved among trees (Gardner et al. 2023). A large stimulation of photosynthesis was associated with a small reduction in stomatal conductance, whereas a weaker photosynthetic response was accompanied by greater stomatal closure (Gardner et al. 2023). These responses can increase intrinsic water‐use efficiency while also altering the gradient of CO_2_ from the atmosphere to the chloroplast. Thus, elevated CO_2_ modifies photosynthesis not only through biochemical effects on carboxylation and photorespiration, but also through changes in the diffusion pathway supplying CO_2_ to Rubisco.

The impact of warming on stomatal conductance is more difficult to resolve because temperature and vapor pressure deficit (VPD) are strongly coupled. Temperature can directly influence stomatal behavior (Urban et al. 2017a, 2017b), but warming also increases the moisture‐holding capacity of air and thereby increases evaporative demand (Campbell and Norman 2000). As VPD rises, reductions in stomatal conductance can limit CO_2_ diffusion into the leaf (Ball et al. 1987), imposing an indirect constraint on photosynthesis that is distinct from the direct biochemical effects of temperature on Rubisco kinetics or RuBP regeneration.

Stomatal conductance also affects photosynthesis through leaf energy balance. Plants efficiently absorb solar radiation to supply the energy required for photosynthesis, but in full sunlight the energy absorbed by leaves exceeds the requirement for photochemistry (Zhu et al. 2010). Excess absorbed energy must be dissipated to minimize lethal leaf temperatures (Marchin et al. 2022; Zahra et al. 2023). Transpiration is a major pathway of energy dissipation as it lowers leaf temperature. Thus, reductions in stomatal conductance under elevated CO_2_, high VPD, or soil water limitation can decrease evaporative cooling and increase leaf temperature (Bernacchi et al. 2007). This feedback can have a direct impact on photosynthesis as biochemical processes are governed by leaf rather than air temperature.

### 4.2 Temperature Response of Mesophyll Conductance

Mesophyll conductance represents the final diffusive step in the pathway from atmospheric CO_2_ to Rubisco, determining the chloroplastic CO_2_ concentration that influences the relative rates of carboxylation and oxygenation. Although the concept of mesophyll conductance was recognized (Ludlow and Wilson 1971), it was assumed to be sufficiently high that it can be ignored in models (von Caemmerer et al. 2025). Subsequent methodological advances, particularly carbon isotope discrimination and combined gas exchange‐chlorophyll fluorescence approaches, demonstrated that mesophyll conductance can limit photosynthesis and alter estimates of key biochemical parameters (Flexas et al. 2008).

Building upon efforts to improve the in vivo temperature responses of key photosynthesis model parameters (Bernacchi et al. 2001, 2003), combined gas exchange and chlorophyll fluorescence measurements were employed to determine whether mesophyll conductance varies with temperature (Bernacchi et al. 2002). This analysis showed that mesophyll conductance is temperature sensitive and can impose a limitation on photosynthesis that ranges from 10% to more than 20% as leaf temperature rises from 10°C to 40°C (Bernacchi et al. 2002). Further evidence showed that mesophyll conductance can vary widely among species and plant functional types, and can respond to environmental and developmental factors including CO_2_, light, water stress, nitrogen availability, and leaf age (Evans 2021; Flexas et al. 2008; Shrestha et al. 2019). These responses occur over both short and long time scales, indicating that mesophyll conductance is likely influenced by leaf structure, membrane properties, aquaporin activity, carbonic anhydrase activity, and/or growth environment (Evans 2021; Flexas et al. 2008; Shrestha et al. 2019).

**FIGURE 4:**
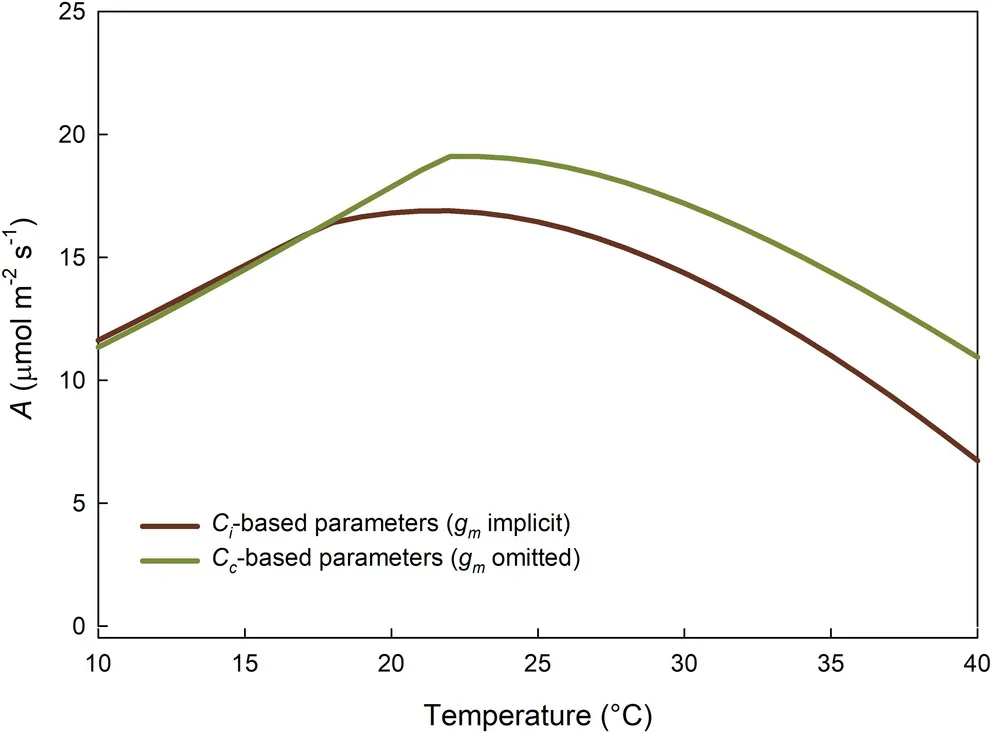
Modeled light‐saturated net CO_2_ assimilation (*A*) versus leaf temperature using *C*_*i*_‐based apparent parameters that implicitly include mesophyll conductance (*g*_*m*_ implicit; Bernacchi et al. 2001) and *C*_*c*_‐based parameters that omit internal CO_2_ diffusion (*g*_*m*_ omitted; Bernacchi et al. 2002). Omitting mesophyll conductance overestimates *A* at high temperatures when Rubisco is limiting.

Incorporating mesophyll conductance into photosynthesis models is essential for separating biochemical responses from potential changes in internal CO_2_ diffusion. Models formulated using *C*_*i*_ and parameterized with apparent kinetic parameters derived from *C*_*i*_‐based gas‐exchange measurements maintain internal consistency between parameter estimation and model application (e.g., Bernacchi et al. 2001). However, these apparent parameters incorporate the influence of mesophyll conductance and may not be transferable across temperatures, species, or growth environments when mesophyll conductance varies. Models parameterized using *C*_*c*_‐based biochemical parameters require explicit representation of mesophyll conductance to calculate CO_2_ concentration at the site of carboxylation (e.g., Bernacchi et al. 2002). For example, modeling photosynthesis using *C*_*c*_‐based parameters (e.g., those derived in vitro or by Bernacchi et al. 2002) without explicitly incorporating mesophyll conductance can significantly overestimate *A* at higher temperatures when Rubisco is limiting (Figure 4). Model performance under elevated CO_2_ and warming depends both on accurate biochemical parameterization and on how stomatal and mesophyll conductance jointly determine *C*_*c*_ across temperatures, species, and growth environments.

Despite advances, mesophyll conductance remains difficult to quantify because fluorescence‐based, isotope‐based, and curve‐fitting approaches rely on different assumptions and can yield different absolute estimates, particularly under stress or rapidly changing environmental conditions (Flexas et al. 2008). Development of an empirically based model of mesophyll conductance analogous to commonly used stomatal‐conductance models (Ball et al. 1987; Leuning 1995), or ideally a mechanistically based model, would help quantify the modeling errors introduced by variation among species and environments. Such a model would also allow the consequences of temperature‐dependent mesophyll conductance for the magnitude and thermal optimum of the CO_2_ response to be tested explicitly.

Together, advances in understanding stomatal and mesophyll conductance show that the CO_2_ × temperature interaction cannot be interpreted solely from atmospheric CO_2_ and biochemical capacity. The response of photosynthesis depends on the CO_2_ concentration at the chloroplast, which is jointly regulated by stomatal aperture, mesophyll conductance, leaf temperature, and environmental conditions. Subsequent work after Long (1991) showed that the original analysis was mechanistically sound but incomplete without explicit representation of *C*_*c*_, stomatal feedbacks, mesophyll conductance, and leaf energy balance. Incorporating these diffusive limitations has improved photosynthesis models and highlights the continuing challenge associated with the range of conductance responses among species, growth environments, and time scales.

## 5 Scaling Responses From Leaf to Canopy

Long (1991) identified that the interaction between CO_2_ and temperature scales beyond the leaf and that scaling to the canopy is not a simple multiplication of leaf‐level responses. Carbon gain at the canopy scale integrates photosynthesis across leaves that differ in light environment, temperature, stomatal conductance, biochemical capacity, and position within the canopy. As a result, the CO_2_ × temperature response depends on the dynamic influences of irradiance, leaf temperature, atmospheric demand for water vapor, canopy structure, and photosynthetic acclimation. Empirical tests of this interaction at the whole‐canopy scale remain limited. Using whole‐tree chambers, Duursma et al. (2014) measured the temperature response of whole‐canopy photosynthesis in trees grown under ambient and elevated CO_2_ and showed that elevated CO_2_ increased the thermal optimum of canopy photosynthesis, broadly supporting the prediction of Long (1991). A broader range of field experiments has imposed warming treatments, frequently in combination with elevated CO_2_, across diverse species and ecosystems (Adachi et al. 2014; Cai et al. 2018; Köhler et al. 2017; Kumar et al. 2020; Ruiz‐Vera et al. 2013, 2015; Yuan et al. 2021), as reviewed by Bernacchi et al. (2023). Incorporating these empirical responses into mechanistically‐based canopy models provide a framework for resolving the complexity of within‐canopy microclimatic dynamics by coupling leaf photosynthesis with canopy radiation transfer, energy balance, water vapor exchange, and vertical gradients in photosynthetic capacity (Drewry et al. 2010a, 2010b; Lochocki et al. 2022; Matthews et al. 2022). Coupling models with empirical field data allows leaf biochemical parameters, canopy properties, meteorological forcing, and seasonal dynamics to be used to test whether leaf‐level responses to elevated CO_2_ and warming translate into proportional changes in canopy carbon gain (Bagley et al. 2015; Bernacchi et al. 2013; Kumagai et al. 2022).

Translating the CO_2_ × temperature interaction from instantaneous leaf responses to daily carbon gain requires integration across dynamic microenvironmental conditions. The leaf photosynthesis model is well suited to this task because it predicts photosynthesis as a function of biochemical capacity and environmental conditions (Farquhar et al. 1980, 2001), but its canopy‐scale expression depends strongly on the timing and location of those conditions. Leaves may operate below, near, and above their thermal optima within the same day and at different locations within the canopy at the same time (Jones 2014). Warming can increase carbon gain during cooler periods or in cooler canopy positions, but reduce photosynthesis when leaf temperature exceeds the optimum or when high VPD induces stomatal limitation (Grossiord et al. 2020). Elevated CO_2_ can shift the photosynthetic thermal optimum upward and suppress photorespiration, as predicted by Long (1991), but the magnitude of this benefit depends on whether elevated CO_2_, light, and leaf temperatures that optimize carbon gain coincide.

Seasonal integration adds further complexity because photosynthetic parameters, canopy development, and meteorological conditions change through time. By incorporating measured acclimation of *V*_*c,max*_ and *J*_*max*_ into leaf and canopy models across two growing seasons, the theoretical CO_2_ × temperature response was evaluated under field conditions (Bagley et al. 2015). The results supported the prediction that elevated CO_2_ and warming can interact synergistically to increase carbon uptake. However, photosynthetic acclimation can reduce leaf‐level photosynthesis across treatments, and models that omitted acclimation overestimated carbon gain under warming but not elevated CO_2_ (Bagley et al. 2015). Seasonal canopy responses were influenced both by instantaneous leaf physiology and by LAI dynamics, meteorological variation, and the timing of warming relative to canopy development. Thus, the CO_2_ × temperature response at the canopy scale is determined by the integration of biochemical responses, acclimation, environmental variability, and canopy phenology rather than by a fixed leaf‐level temperature response alone.

Canopy structure further modifies diurnal and seasonal carbon gain by distributing leaves across contrasting light and thermal environments. Long (1991) highlighted the importance of sunlit and shaded leaves, noting that elevated CO_2_ can reduce the light compensation point and increase the contribution of shaded leaves to canopy photosynthesis. Subsequent canopy models with improved computational capabilities and higher‐quality validation data incorporated within‐canopy dynamics by separating direct and diffuse radiation and partitioning several canopy layers into sunlit and shaded fractions (Drewry et al. 2010a). Sunlit canopy layers experience high irradiance, higher leaf temperatures, and greater atmospheric demand for water vapor, whereas shaded leaves operate under lower light and may benefit disproportionately from elevated CO_2_ through reduced photorespiration and lower light compensation points (Drewry et al. 2010a, 2010b). More recent analyses of canopy architecture and energy balance emphasize the role of leaf angle, reflectance, chlorophyll content, and canopy vertical structure in light distribution and leaf temperature (Bernacchi et al. 2025).

At ecosystem scales, canopy‐level processes influence estimates of gross primary productivity and projections of crop productivity, ecosystem carbon balance, and land‐atmosphere feedbacks. The leaf photosynthesis model remains a core component of many canopy, ecosystem, terrestrial biosphere, and Earth system models, but model performance depends strongly on how photosynthetic parameters are assigned and allowed to vary across species, canopy position, leaf age, nitrogen availability, growth environment, and acclimation (Bagley et al. 2015; Bernacchi et al. 2013; Bonan et al. 2011, 2012; Groenendijk et al. 2011; Hickler et al. 2008; Kattge et al. 2009). As a result, model‐data mismatch can arise when leaf‐level parameterizations are applied uniformly across canopies or seasons, when vertical gradients in photosynthetic capacity are assumed rather than measured, or when acclimation and diffusive limitations are omitted (Bagley et al. 2015; Bernacchi et al. 2013; Drewry et al. 2010a, 2010b).

With an increasing number of long‐running micrometeorological experiments, such as eddy covariance measurements (Baldocchi 2003), there is an opportunity to test whether the thermal optimum of ecosystem photosynthesis has changed with rising atmospheric CO_2_. The longest‐running forest eddy covariance site began operating in 1989 (Wofsy et al. 1993), and the longest‐running cropping‐system site began operating in 1996 (Bernacchi et al. 2005). These, and similar, continuous data records, coupled with the rise in atmospheric CO_2_, may provide insights into changes in the thermal optimum of canopy photosynthesis over time. For example, the temperature optimum of canopy photosynthesis across Australian wooded ecosystems was shown to align with air temperature, demonstrating the feasibility of estimating ecosystem‐scale thermal optima from micrometeorological data (Bennett et al. 2021). Newer approaches are increasingly constraining canopy photosynthesis and its underlying parameters, including model‐data assimilation (LeBauer et al. 2013; Wang et al. 2013), remote sensing and solar‐induced fluorescence (Coops et al. 2010; Damm et al. 2010; Frankenberg et al. 2011; Kimm et al. 2021), and hyperspectral or high‐throughput phenotyping approaches for estimating photosynthetic traits under field conditions (Bernacchi et al. 2025; Fu et al. 2022). While some of these approaches may not be able to explicitly resolve a CO_2_ × temperature interaction, they may be able to constrain associated changes in photosynthetic acclimation and canopy architectural responses to environmental conditions that influence ecosystem carbon uptake.

Together, canopy and ecosystem modeling studies show that the biochemical CO_2_ × temperature interaction identified by Long (1991) remains central to predicting photosynthetic carbon gain, but its expression depends on the temporal and spatial integration of physiological, structural, and environmental processes. The interaction persists at the canopy scale, but it is filtered through phenology, canopy architecture, canopy energy balance, source‐sink dynamics, and meteorological variability. Thus, the challenge is no longer whether the CO_2_ × temperature interaction exists, but when, where, and how strongly it is expressed across temporal and spatial scales.

## 6 Evidence‐Based Opportunities for Overcoming Photosynthetic Limitations to Temperature

The predictive logic established by Long (1991) and refined through subsequent experimental validation provides the basis for improving the thermotolerance of photosynthesis. In C_3_ plants, the major biochemical targets include Rubisco activation, photorespiratory metabolism, and RuBP regeneration (Bernacchi et al. 2025; Cavanagh and Matthews 2025). Over the past decade, strategies to improve crop photosynthesis by increasing Rubisco content or catalysis, mesophyll conductance, photoprotection, and electron transport; altering canopy architecture; and modifying photorespiratory metabolism have moved from theoretical prediction to field demonstration in model and agronomic crops (Long et al. 2025). However, field‐based evidence for improved photosynthetic performance under elevated temperature remains limited to modified photorespiration and increased RuBP regeneration capacity.

### 6.1 SBPase Overexpression

Increasing RuBP regeneration requires increasing flux through the regenerative phase of the Calvin–Benson cycle, which converts glyceraldehyde‐3‐phosphate into ribulose‐1,5‐bisphosphate through a series of enzyme‐catalyzed reactions that require ATP from the light reactions. Though early antisense studies showed that control over CO_2_ assimilation is distributed across the cycle rather than determined by a single regenerative enzyme, several enzymes emerged as useful engineering targets, including sedoheptulose‐1,7‐bisphosphatase (SBPase), fructose‐1,6‐bisphosphate aldolase (FBPA), and transketolase (Raines 2003, 2022; Stitt and Schulze 1994). Increased SBPase activity, either through overexpression of SBPase or expression of a cyanobacterial bifunctional SBPase/FBPase, has increased photosynthesis, growth, or biomass across many C_3_ species (Driever et al. 2017; Köhler et al. 2017; Lefebvre et al. 2005; Miyagawa et al. 2001; Simkin et al. 2015; Suzuki et al. 2019). The environmental dependence of this response was tested directly in FACE‐grown tobacco, where SBPase overexpression produced a larger stimulation of photosynthesis and biomass under elevated CO_2_ than in wild‐type plants (Rosenthal et al. 2011). Increased SBPase activity also improved photosynthetic performance under high‐temperature stress in rice, indicating that greater regeneration capacity can sustain carbon assimilation when temperature constrains photosynthetic metabolism (Feng et al. 2007).

Evidence from field‐grown soybean provides the strongest test of this approach under realistic future‐climate conditions. Transgenic soybean overexpressing a cyanobacterial bifunctional FBP/SBPase was grown under combined elevated CO_2_ and canopy warming of +2.7°C (Köhler et al. 2017). Across treatments, transgenic plants maintained higher photosynthetic carbon assimilation, *V*_*c,max*_, and *J*_*max*_ than unmodified control plants. Warming reduced yield across genotypes when grown at ambient CO_2_ concentrations (400 ppm). Under elevated CO_2_ (600 ppm), overexpression of FBP/SBPase was thermoprotective: heated transgenic plants maintained yield equivalent to unheated plants, whereas wild‐type plants showed yield reductions of 11%–22% (Köhler et al. 2017). Together, these results support increased RuBP regeneration as a target for improving photosynthetic performance under future conditions. Despite extensive evidence that Calvin‐Benson cycle enzymes can be engineered to increase photosynthesis (Simkin et al. 2019), only a single RuBP‐regeneration target has been tested under realistic field heating in combination with elevated CO_2_. Mechanistic and multiscale crop modeling is therefore needed to prioritize regenerative enzymes or combinations of enzymes by linking biochemical changes in RuBP regeneration to leaf and canopy assimilation and yield under future temperature conditions (Cavanagh and Matthews 2025; Simkin et al. 2019).

### 6.2 Photorespiratory Bypass

Two major strategies for modifying photorespiratory metabolism have been tested: increasing the capacity of the native photorespiratory pathway and introducing synthetic pathways that metabolize glycolate more efficiently (Cavanagh and Ort 2023; Sun et al. 2024). Native‐pathway approaches increase the capacity of photorespiratory enzymes, including components of the glycine cleavage system, to accelerate recycling of photorespiratory intermediates and reduce metabolic bottlenecks (López‐Calcagno et al. 2019; Prasad et al. 2017; Sun et al. 2024). Synthetic bypasses divert the glycolate produced by Rubisco oxygenation into alternative reactions, often within the chloroplast, reducing metabolite export through the native chloroplast–peroxisome–mitochondrion pathway and increasing the probability that released CO_2_ is refixed by Rubisco (Meacham‐Hensold et al. 2024; Peterhansel et al. 2013). Over the past decade, major advances in engineering have moved from proof‐of‐concept testing in model plants under controlled environments to replicated field trials in tobacco, potato, and rice (Kebeish et al. 2007; Maier et al. 2012; Meacham‐Hensold et al. 2024). Both engineering strategies suggest that growth and physiological benefits are enhanced under high‐temperature conditions when photorespiratory pressure increases, although field evidence for improved performance under elevated temperature is strongest for synthetic bypasses (Cavanagh and Ort 2023; Sun et al. 2024; Timm et al. 2019).

In field‐grown tobacco overexpressing a glycolate oxidation photorespiration bypass, the daily integral of photosynthetic carbon assimilation was 5%–8% higher than controls, resulting in a 19%–37% increase in end‐of‐season biomass (South et al. 2019). To test the impact of bypassing photorespiration on thermal resilience, the same plants were grown under field‐canopy warming of 5°C above ambient temperatures, where they maintained approximately 16% greater daily carbon gain than unmodified controls and reduced the yield penalty associated with warming by 19%. Consistent with increased Rubisco oxygenation at elevated temperature, the benefits of bypassed photorespiration were strongest under experimental heating. Plants expressing a bypass produced 26% more total biomass than heated wild‐type plants, compared with an 11% biomass advantage under ambient conditions (Cavanagh et al. 2022). When transformed into potato, the same pathway increased tuber biomass by 9%–30% across two field seasons without a decline in nutritional quality. The greatest yield increases were observed in a growing season characterized by two naturally occurring heatwaves, when bypass‐expressing plants produced 30% more tuber biomass and maintained higher photosynthetic capacity during bulking, including up to 23% higher *V*_*cmax*_ and 13% higher *J*_*max*_ (Meacham‐Hensold et al. 2024).

Together, field trials demonstrate that the biochemical limitations identified by Long (1991) can be modified in intact crop canopies. Increasing RuBP regeneration protects photosynthesis and yield under combined elevated CO_2_ and warming, while photorespiratory bypasses reduce the carbon penalty of oxygenation and improve biomass accumulation under field warming. These strategies do not remove the need to account for stomatal conductance, mesophyll conductance, canopy energy balance, acclimation, or source–sink constraints. They do, however, show how understanding the biochemical limitations underpinning the CO_2_ × temperature interaction can generate experimental targets. The remaining challenges are to test other promising engineering targets under future‐stress conditions and to determine whether these gains persist across crop species, developmental stages, growing seasons, and the frequency and intensity of heat events expected under future climates.

## 7 Conclusions and Future Directions

Long (1991) provided a mechanistic explanation for why rising CO_2_ and increasing temperature cannot be treated as independent drivers of photosynthesis. By linking the temperature dependence of Rubisco carboxylation and oxygenation to the suppression of photorespiration under elevated CO_2_, his analysis showed that CO_2_ enrichment modifies both the magnitude of photosynthetic carbon gain and the shape of the photosynthetic temperature response. Subsequent research has largely validated this prediction while also revealing that the realized response depends not only on Rubisco biochemistry but also on RuBP regeneration, photosynthetic acclimation, stomatal and mesophyll diffusion, leaf energy balance, canopy structure, phenology, and environmental constraints.

A major legacy of Long's work is that it defined the assumptions that needed testing to improve the model's ability to predict plant responses to climate change. Advances in gas exchange, chlorophyll fluorescence, enzyme parameterization, and field experimentation refined the temperature responses of *V*_*c,max*_ and *J*_*max*_ and showed that these parameters are dynamic rather than fixed. Growth under elevated CO_2_, warming, or their combination was shown to alter Rubisco content, Rubisco activation state, electron transport capacity, and the balance between carboxylation and RuBP regeneration. Stomatal and mesophyll conductance measurements showed how much of the atmospheric CO_2_ signal reaches the chloroplast and how changes in CO_2_, temperature, vapor pressure deficit, and water availability modify photosynthesis. Furthermore, the biochemical interaction between CO_2_ and temperature varies across space and time. Leaves within a canopy do not experience the same environment or operate at the same point on the photosynthetic temperature response curve. The effect of elevated CO_2_ and warming on canopy carbon gain depends on when favorable CO_2_, light, temperature, and water conditions coincide. Models have advanced beyond the formulation utilized by Long (1991) but still require a more dynamic representation of photosynthetic capacity, acclimation, diffusion, and canopy energy balance across developmental and environmental gradients.

Field evidence that enhanced RuBP regeneration or photorespiratory bypasses can improve carbon gain, biomass, or yield under elevated CO_2_ and warming demonstrates that the biochemical constraints underlying the CO_2_ × temperature interaction can be modified in intact crop canopies. Future work should test additional targets (as outlined in Bernacchi et al. 2025) and combinations of targets under realistic field conditions, including elevated CO_2_, global warming, extreme heat events, high vapor pressure deficit, and variable water or nutrient supply. These efforts should be evaluated across scales, linking biochemical modifications to leaf photosynthesis, canopy carbon gain, water use, and, for agronomically important species, yield.

Since Long (1991), the central question has shifted from whether elevated CO_2_ modifies the temperature response of C_3_ photosynthesis to how much this interaction affects carbon gain under realistic environmental conditions, and whether natural variation or targeted photosynthetic improvements can be used to strengthen crop performance in a changing climate. Long (1991) provided the conceptual foundation by showing that photosynthetic responses to climate change could be understood mechanistically from the entry point of carbon into the biosphere. The continuing challenge is to extend that understanding from Rubisco to leaves, canopies, ecosystems, and crops in ways that improve prediction and guide adaptation in a warmer and CO_2_‐enriched world.

## Author Contributions

**Carl J. Bernacchi:** conceptualization, funding acquisition, writing – original draft, writing – review and editing, formal analysis, supervision, methodology. **Amanda P. Cavanagh:** conceptualization, funding acquisition, writing – original draft, writing – review and editing, formal analysis, supervision, methodology.

## Funding

This work was supported by Gates Agricultural Innovations (grant investment 57248).

## Conflicts of Interest

The authors declare no conflicts of interest.

## Data Availability

Data sharing not applicable to this article as no datasets were generated or analysed during the current study.
